# A Study on the Road Performance of the Self-Healing Microcapsule for Asphalt Pavement

**DOI:** 10.3390/ma18153483

**Published:** 2025-07-25

**Authors:** Pei Li, Rongyi Ji, Chenlong Zhang, Jinghan Xu, Mulian Zheng, Xinghan Song

**Affiliations:** 1 Guangdong Road and Bridge Construction Development Co., Ltd., Guangzhou 510635, China; lipeijiaoer@163.com (P.L.); zhchl1987@126.com (C.Z.); 2 Key Laboratory for Special Area Expressway Engineering of Ministry of Education, Chang’an University, Xi’an 710064, China; jirongyi@chd.edu.cn (R.J.); 2021021051@chd.edu.cn (J.X.); songxinghan@chd.edu.cn (X.S.)

**Keywords:** asphalt pavement, micro-cracks, microcapsule, road performance, self-healing property, epoxy resin, two-component

## Abstract

Asphalt pavement cracking is an important factor affecting its service life. Under certain conditions, the self-healing behavior of asphalt itself can repair pavement cracks. However, the self-healing ability of asphalt itself is limited. In order to strengthen the self-healing ability of asphalt, the microcapsule wrapped with a repair agent is pre-mixed into the asphalt mixture. When the crack occurs and spreads to the surface of the microcapsule, the microcapsule ruptures and the healing agent flows out to realize the self-healing of the crack. Current microcapsules are mostly prepared with healing agents and bio-oil as core materials, and their high-temperature resistance to rutting is poor. While the epoxy resin contains a three-membered cyclic ether, it can undergo ring-opening polymerization to bond and repair the asphalt matrix. In addition, research on microcapsules mainly focuses on the self-healing properties of microcapsule-modified asphalt. In fact, before adding microcapsules to asphalt to improve its self-healing performance, it is necessary to ensure that the asphalt has a good road performance. On this basis, the self-healing performance of asphalt is improved, thereby extending the service life of asphalt pavement. Therefore, two-component epoxy self-healing microcapsules (E-mic and G-mic) were first prepared in this paper. Then, a temperature scanning test, rheological test of bending beams, and linear amplitude scanning test were, respectively, conducted for the microcapsule/asphalt to evaluate its road performance, including the high-temperature performance, low-temperature crack resistance, and fatigue performance. Finally, the self-healing performance of microcapsules/asphalt was tested. The results showed that the self-developed epoxy self-healing microcapsules were well encapsulated and presented as spherical micron-sized particles. The average particle size of the E-mic was approximately 23.582 μm, while the average particle size of the G-mic was approximately 22.440 μm, exhibiting a good normal distribution. In addition, they can remain intact and unbroken under high-temperature conditions. The results of road performance tests indicated that the microcapsule/asphalt mixture exhibits an excellent high-temperature resistance to permanent deformation, low-temperature crack resistance, and fatigue resistance. The self-healing test demonstrated that the microcapsule/asphalt exhibited an excellent self-healing performance. When the microcapsule content was 4%, the self-healing rate reached its optimal level of 67.8%, which was 149.2% higher than that of the base asphalt.

## 1. Introduction

Asphalt pavement can repair micro-cracks and local damage by its self-healing ability under normal temperatures and in service conditions. While, the self-healing ability is limited and the speed is very slow [1,2], many researchers proposed using additives to enhance the self-healing ability. Diao et al. demonstrated that the fatigue self-healing efficiency of asphalt concrete could be improved by using slaked lime as a filler [3,4,5]. Zhang et al. found that molecular and nano-level polymers could enhance the self-repairing property of asphalt mixtures [6]. Jin et al. studied the impact of adding self-healing microcapsules on asphalt performance. Their research results showed that incorporating microcapsules into asphalt can effectively enhance its self-healing properties. As the number of microcapsules increases, the self-healing index of microcapsule-containing asphalt first increases and then decreases [7]. Ding et al. established a macrostructure model for asphalt pavement and a multiscale model of the representative volume element (RVE) incorporating self-healing microcapsules using the finite difference method. They systematically analyzed the effects of the microcapsule content, capsule wall modulus, and capsule wall thickness on the mechanical properties of different asphalt pavements. The results indicate that the microcapsule content, microcapsule wall material modulus, and wall membrane thickness have a significant impact on the road performance and self-healing ability of asphalt pavement [8]. After developing self-healing microcapsules, Wang et al. analyzed the impact of microcapsules on the self-healing and fatigue life of asphalt and found that when the dosage was 2%, the improvement effect was better [9]. Zhang et al. synthesized MMF microcapsules through in situ polymerization and analyzed the improvement effects of adding the microcapsules on the temperature sensitivity, self-healing ability, and fatigue life of asphalt [10].

While microcapsule-modified asphalt significantly improves the self-healing efficiency and fatigue life of asphalt, its incorporation may exert complex impacts on other pavement performances of asphalt [11,12,13,14]. Wen et al. prepared microcapsules with different core–shell ratios by coating soybean oil and melamine–urea–formaldehyde (MUF). The microcapsules were characterized by a particle size analysis, a morphology observation, and other methods to determine the optimal core–shell ratio. The results showed that the microcapsules had a good particle state when the core–shell ratio was 1:2. The viscosity of asphalt increases after the incorporation of microcapsules. Upon the rupture of the microcapsules, the release of the soybean oil alters the composition ratio of the asphalt, leading to a decrease in the high-temperature performance. However, the remaining microcapsule shells continue to enhance the deformation resistance of asphalt [15]. Song et al. prepared urea–formaldehyde–epoxy resin (UFE) microcapsules using polyurea formaldehyde as the wall material and epoxy resin as the core material and concluded that UFE improved both the high-temperature deformation resistance and self-healing ability of SBS-modified asphalt [16]. Wang and his team discovered that modifying the microcapsule shell with 1 wt% graphene improved the high-temperature rutting resistance of asphalt, indicating that microcapsules containing graphene are beneficial [17]. However, the incorporation of microcapsules has a negative impact on the low-temperature performance of asphalt. When the content of microcapsules is too high, the ductility of the modified asphalt will be unqualified [10,18]. In order to improve the low-temperature crack resistance of the asphalt binder, researchers usually modify microcapsules. Wen et al. adopted carbon nanotube-modified microcapsules [19], and Ren et al. adopted graphene-modified microcapsules [20]. It was found that the low-temperature performance of the microcapsule-modified asphalt was improved.

At present, there are mainly three kinds of materials used as the core materials of microcapsules for asphalt, including asphalt-based materials, biological oil (Bio-oil), and adhesive materials [21,22,23]. Asphalt-based materials mainly included an asphalt rejuvenator and a light oil [24,25]. Asphalt-based materials have a poor high-temperature performance. When they are applied to pavement, their resistance to permanent deformation becomes worse, and the fatigue life decreases. Biological oil belongs to environmentally friendly materials, which are conducive to energy conservation and environmental protection to reduce emissions. However, its viscosity is low, and the fluidity is high, resulting in easy overflows under high temperatures [26]. Adhesive materials mainly include epoxy resin and waterborne epoxy resin. Adhesive materials repair micro-cracks through the curing and bonding effect, which can be regarded as the repair of cracks in the real sense. Nowadays, the molecular structure of the ordinary epoxy resin used contains a significant amount of unsaturated bonds, which are easy to degrade under ultraviolet radiation and have a poor anti-aging performance [27]. The current research on microcapsule asphalt mainly focuses on self-healing properties. The incorporation of microcapsules into asphalt can be considered as a modification of the material itself. The prerequisite for enhancing the self-healing performance of asphalt is to have an ideal road performance, at least not sacrificing other performances.

**Figure 1:**
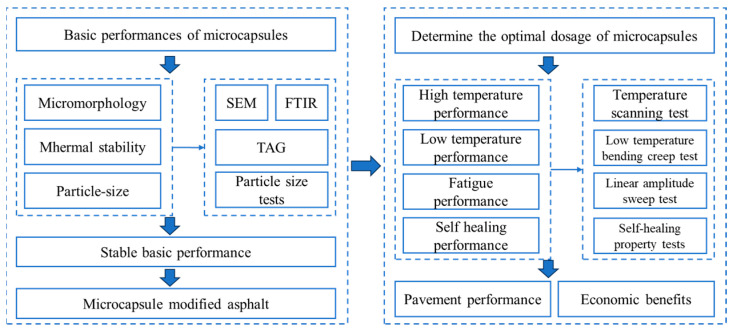
The research process of this paper.

In this paper, two-component microcapsules, E-mic and G-mic, were first prepared using modified epoxy resin E and curing agent G, respectively, as the core materials of the microcapsules, both of which exhibit excellent weather resistance. Secondly, the microcapsules prepared were added to the asphalt to prepare the microcapsules/asphalt material. Meanwhile, temperature scanning, a low-temperature bending creep test, and a linear amplitude scanning test were conducted to evaluate the road performance of the microcapsule/asphalt. Finally, the self-healing property test was performed to evaluate the self-healing property of the microcapsule/asphalt. The research process is illustrated in Figure 1.

## 2. Test Materials and Methods

### 2.1. Test Materials

#### 2.1.1. Asphalt

The asphalt adopted the Korean SK70 base asphalt. The technical indicators are shown in Table 1.

#### 2.1.2. Microcapsules

(1) Core Material

**Figure 2:**
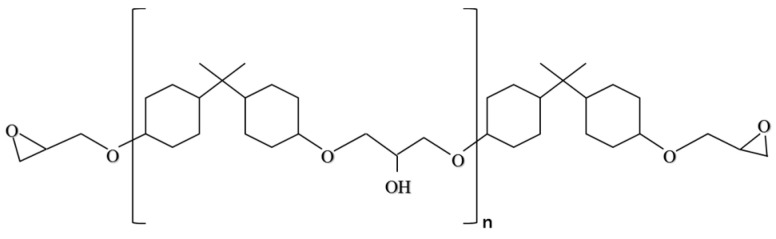
The chemical structure of E. Reprinted with permission from Ref. [23]. Copyright 2020 Elsevier.

Ordinary epoxy resin materials contain a large number of unsaturated linkages, which will be cracked under ultraviolet light. Based on this, epoxy resin E with better aging resistance and weather resistance was selected as the epoxy component of epoxy self-healing microcapsules. Epoxy resin E is a colorless and transparent low-viscosity liquid. It is obtained by modifying ordinary bisphenol A epoxy resin under high-temperature and high-pressure conditions. The molecule of E is a six-membered ring without double bonds and unsaturated bonds and has an extremely low chlorine content. Its structural formula is shown in Figure 2. E can be cured under low-temperature, medium-temperature, and UV light conditions, and its cured product has strong aging resistance and weather resistance. Its main technical indicators are listed in Table 2.

**Figure 3:**
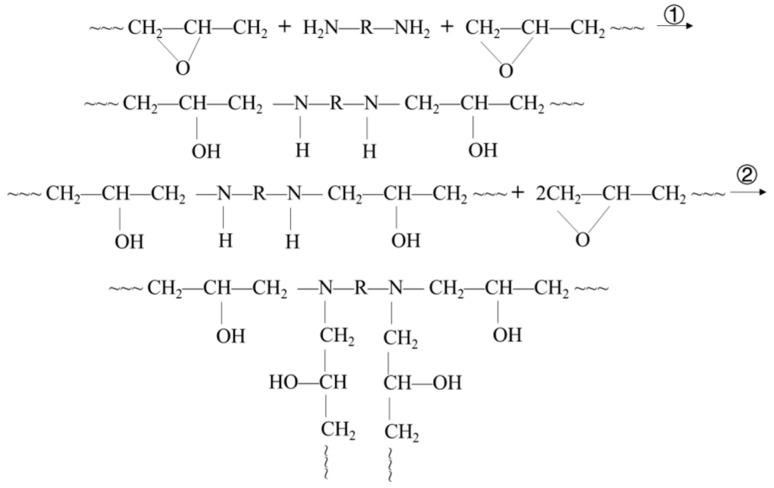
Curing reaction of epoxy resin E and curing agent G.

A medium-to-low temperature curing agent G, based on a mixed modified amine system, was selected as the curing agent. Its main components include triethylenetetramine (TETA) and diethylenetriamine (DETA), etc. It can initiate curing reactions at temperatures above 0 °C and effectively repair pavement damage within the typical service temperature range of pavements. The curing process proceeds in two steps: first, the epoxy groups of the epoxy resin open and undergo grafting reactions with the curing agent to form hydroxyl groups; second, the grafted products further crosslink with unreacted epoxy groups, eventually forming a three-dimensional network structure. The specific reaction process is shown in Figure 3.

(2) Shell Material

**Figure 4:**
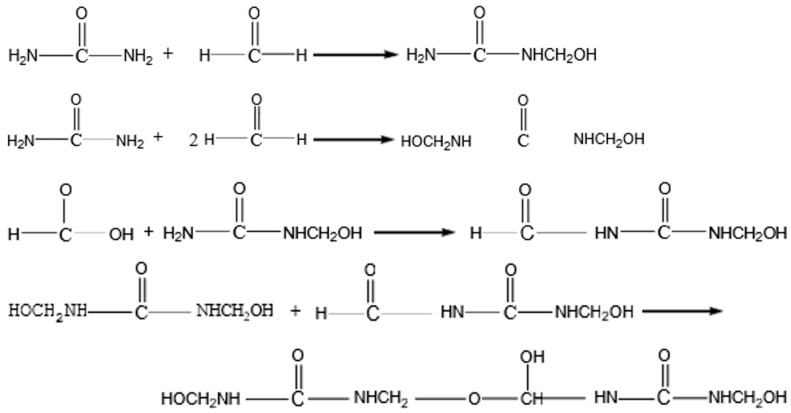
Synthetic reaction of MUF.

Common non-biodegradable polymer shell materials for microcapsules include melamine formaldehyde resin (HMMM), melamine modified urea–formaldehyde resin (MUF), etc. Among them, HMMM, despite its excellent chemical stability, tends to react prematurely with the core material epoxy resin E, which may cause microcapsule failure. In contrast, MUF not only has lower cost but also exhibits good thermal stability and mechanical strength. In this study, MUF was selected as the shell material for microcapsules. Table 3 lists the raw materials of MUF. The pre-polymer can produce condensation polymerization reaction with an acidic catalyst and at a certain temperature, thus forming the polymer MUF. This reaction is shown in Figure 4.

(3) Preparation of Microcapsules

**Figure 5:**
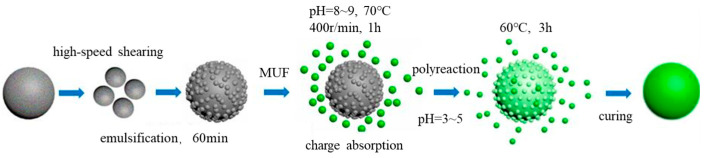
Microencapsulation process. Reprinted with permission from Ref. [23]. Copyright 2020 Elsevier.

To prepare microcapsules, the first step is to prepare the shell material (MUF) of the microcapsules. Subsequently, the core material (E/G) was emulsified. Afterwards, the MUF was added into the core material emulsion drop by drop. And then, the curing reaction was conducted for 3 h after adding the catalyst, facilitating the curing reaction. Finally, the microcapsules were obtained after washing with distilled water and filtering. The process is shown in Figure 5.

#### 2.1.3. Microcapsule/Asphalt

Asphalt was heated to 160 °C ± 5 °C, and microcapsules E-mic and G-mic were sequentially added to the asphalt. The microcapsule/asphalt was stirred with a blender at 500 r/min for 30 min to prevent uneven microcapsule particle agglomeration and dispersion. The samples were prepared at the dosages of 1%, 2%, 3%, and 4%, respectively, and the dosage was the mass ratio of E-mic to asphalt (the mass ratio of E-mic to G-mic was 2:1). Therefore, the four groups of microcapsule/asphalt samples were, respectively, denoted by 1% E-mic, 2% E-mic, 3% E-mic, and 4% E-mic in this study.

### 2.2. Test Methods

#### 2.2.1. Performance Tests of the Microcapsule

The microstructure and thermal stability of the microcapsule will directly affect its performance in asphalt and asphalt mixture. In this study, micromorphology, particle size distribution, and thermal stability tests were carried out, respectively.

(1) Micromorphology and Microstructure Tests

Micromorphology and microstructure of the microcapsules were observed by SEM. The SEM was produced by Carl Zeiss AG, Jena, Germany (model MERLIN Compact). Before conducting observations, it was necessary to immobilize the microcapsule powder and endow it with conductivity (by spraying gold on its surface). Subsequently, an electron beam scanning observation was performed with a scanning voltage of 5 kV and a current of 10 μA, thereby obtaining the surface micromorphology and microstructure of the microcapsules.

(2) Particle Size Tests

The instrument used for particle size distribution testing was sourced from the UK and was modeled as the Nastersizer 2000 (Malvern Panalytical, Malvern, UK). The dispersing agent was ethanol with a refractive index of 1.330, particle absorptivity of 0.100, and particle refractive index of 1.587. The scattering model was Mie, the analysis model was universal, and the analysis sensitivity was set to enhancement.

(3) Thermogravimetric Analysis (TGA) Tests

Microcapsules used for modifying asphalt must possess good high-temperature stability to ensure that most of the capsules remain intact after high-temperature mixing and construction. Therefore, TGA is used to test the state of microcapsules at high temperatures. The Synchronous thermal analyzer adopted is produced in America, and its model is Discovery SDT 650 (TA Instruments, New Castle, DE, USA).

About 10 mg microcapsule samples were weighed and loaded into alumina crucible, and the heating process was carried out in an N_2_ flow of 40 mL. A stepwise isothermal heating mode was adopted in order to capture the mass change more accurately. The heating rate was 10 °C·min^−1^, and the heating range was 40~600 °C.

(4) Fourier Transform Infrared Spectroscopy (FTIR) Tests

Infrared absorption spectroscopy images can reveal the functional group information of a substance. By utilizing FTIR to test the changes in functional groups before and after asphalt modification, one can infer the chemical reactions that occurred during the modification process [28,29,30]. The test was conducted using a Tensor II infrared spectrometer produced in Germany. The resolution was set to 4 cm^−1^, and the test wavenumber ranged from 400 to 4000 cm^−1^.

#### 2.2.2. Temperature Scanning (TS) Test

Temperature scanning test was used to analyze the high-temperature rheological properties of microcapsule/asphalt to evaluate its resistance to permanent deformation at high temperature. The samples were tested using a SmartPave 102 (Anton Paar, Graz, Austria). A parallel plate of 8 mm was used with a 2 mm plate spacing. The tests adopted strain control mode, and the strain was set at 12%. The angular frequency was set at 10 rad/s. The test temperature was set at 52 °C, 58 °C, 64 °C, and 70 °C.

#### 2.2.3. Low-Temperature Bending Creep Stiffness Test

The low-temperature bending creep stiffness test was used to evaluate the cracking resistance of microcapsules/asphalt at low temperature. The low-temperature bending beam rheometer (BBR) is produced in the United States, model TE-BBR-F. The samples were prepared as asphalt beams with a size of 127 mm × 12.7 mm × 6.35 mm. Three parallel samples were prepared for each condition. The beam should be preloaded to ensure its close contact with the bracket. Afterwards, a constant load of 980 mN ± 25 mN was applied to the beam for 240 s at a given temperature. Meanwhile, the central deformation of the beam was measured by a displacement sensor. The creep stiffness modulus (S) and the creep velocity (m) of the beam at 8.0 s, 15.0 s, 30 s, 120 s, and 240 s were recorded to analyze the cracking resistance of microcapsules/asphalt at low temperature. The test temperature was set to −12 °C, −18 °C, and −24 °C.

#### 2.2.4. Linear Amplitude Sweep (LAS) Test

The linear amplitude sweep (LAS) tests were used to analyze the effect of microcapsules on the fatigue resistance of matrix asphalt. The same DSR as in the previous TS test was employed for this test. PAV aging was performed on the prepared microcapsule/asphalt before sample preparation. The samples prepared were 8 mm in diameter and 2 mm in spacing, and the test temperature was set at 25 °C. The LAS test consists of two steps according to AASHTO TP 101-14 [31]. The first step is to analyze the rheological properties of samples by frequency scanning. The frequency scanning adopts the controlled strain mode, and the strain is set to 0.1%. The test frequency is set to 0.2, 0.4, 0.6, 0.8, 1.0, 2.0, 4.0, 6.0, 8.0, 10, 20, and 30 Hz. The complex modulus and phase angle at each frequency are recorded during the tests. The second step is to perform an amplitude scanning test. The test frequency (*ω*) is set at 10 Hz, and the sinusoidal load amplitude is linearly increased from 0.1% to 30%. During the test, the time interval of data recording is 1 s. The amplitude scanning test is loaded for 3100 cycles with a duration of 310 s.

After the frequency scanning test, the storage modulus *G*′(*ω*) was obtained. log *ω* was used as the horizontal axis and log *G*′(*ω*) as the vertical axis, and linear fitting was performed by Equation (1). Then, parameter α can be calculated by Equation (2).

(1) $$\log G^{\prime }\left(\omega \right)=m\left(\log\omega \right)+b$$

(2) α=1/m

The accumulated damage of the sample after the amplitude scanning test can be calculated by Equation (3).

(3) $$D(t)≅\sum_{i=1}^{N}{\left[\pi \gamma_{0}^{2}\left(C_{i-1}-C_{i}\right)\right]}^{\alpha /1+\alpha }{\left(t_{i}-t_{i-1}\right)}^{1/1+\alpha }$$

where $\left|G*\right|$ denotes complex shear modulus (MPa); $\gamma_{0}$ denotes the strain at a given point (%); *t* denotes test time (s); $C(t)=\left|G^{*}\right|(t)/{\left|G^{*}\right|}_{initial}$ is the $\left|G*\right|$ at time t divided by the initial undamaged $\left|G*\right|$ (MPa). The value of the $\left|G*\right|$ is the value of the second point, because the modulus of the first point after changing the condition from the static state was different from the undamaged modulus at the target loading frequency. At time *t*, C(t) and D(t) are recorded, assuming that *C* = 1 when D(0)=0. The relationship between $\left|G*\right|\sin\delta$ and D(t) is shown in Equation (4).

(4) $$C(t)=C_{0}-C_{1}{\left(D\right)}^{C_{2}}$$

where $C_{0}=1$. $C_{1}$ and $C_{2}$ are the coefficients derived from curve fitting using Equation (5).

(5) $$\log\left(C_{0}-C(t)\right)=\log\left(C_{1}\right)+C_{2}\cdot \log\left(D(t)\right)$$

$D_{f}$ denotes the D(t) corresponding to fatigue damage, and can be calculated by Equation (6).

(6) $$D_{f}={\left(\frac{C_{0}-C_{at\;Peak\;Stress}}{C_{1}}\right)}^{1/C_{2}}$$

Parameters A and B of the fatigue damage model of asphalt can be calculated by Equation (7).

(7) $$A=\frac{f{\left(D_{f}\right)}^{k}}{k{\left(\pi C_{1}C_{2}\right)}^{\alpha }}$$

where *f* denotes loading frequency (10 Hz); *k* = 1 + (1 − *C*_2_)*α*; and *B* = 2*α*.

Fatigue performance parameter $N_{f}$ of asphalt can be expressed in Equation (8).

(8) $$N_{f}=A{\left(\gamma_{\max}\right)}^{-B}$$

where $\gamma_{\max}$ denotes the maximum strain of a given pavement structure (%).

#### 2.2.5. Self-Healing Property Tests

**Figure 6:**
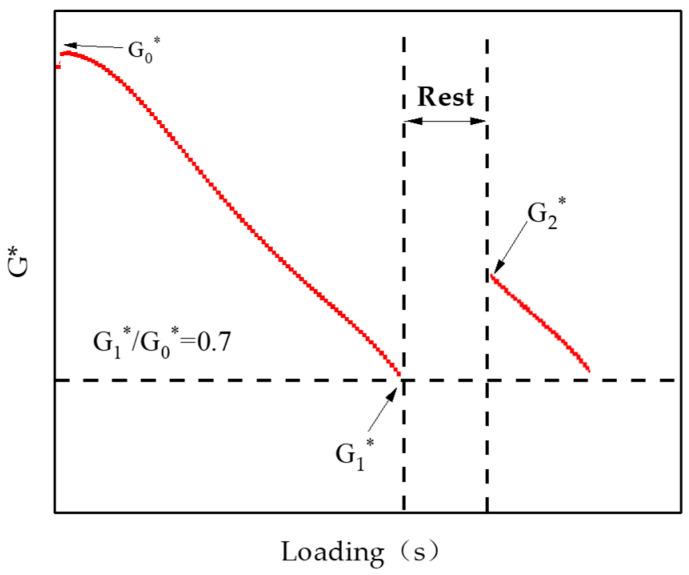
A schematic diagram of the “Fatigue–Rest–Fatigue” test.

“Fatigue–Rest–Fatigue” test based on DSR was designed to evaluate the self-healing property of microcapsules/asphalt in this study. A parallel plate of 8 mm was used with a 2 mm plate spacing. The loading frequency was set at 10 Hz. The stress was set to 3%, and the temperature was set at 25 °C. Relevant studies have shown that the complex shear modulus (G*) drops to 70% of the initial modulus ($G_{0}^{*}$), which can better represent the self-healing performance of asphalt materials [32]. Therefore, in this experiment, the loading was stopped when G* dropped to 70% of $G_{0}^{*}$, completing the first “fatigue” test. Subsequently, the specimens were kept at 25 °C for 1 h, that is, the “rest” period to repair the fatigue damage. Afterwards, tests continued until G* dropped again to 70% of $G_{0}^{*}$. It was the second “fatigue” test. “Fatigue–Rest–Fatigue” test is illustrated in Figure 6.

The self-healing rate of microcapsule/asphalt samples can be calculated by Equation (9).

(9) $$SH=\frac{G_{2}^{*}-G_{1}^{*}}{G_{0}^{*}-G_{1}^{*}}\times 100\%$$

where SH denotes the self-healing rate of microcapsule/asphalt; $G_{0}^{*}$ denotes the initial complex shear modulus; $G_{1}^{*}$ denotes the complex shear modulus before the repair period; $G_{2}^{*}$ denotes the complex shear modulus after the repair period.

## 3. Results and Discussions

### 3.1. Basic Performances of Microcapsules

**Figure 7:**
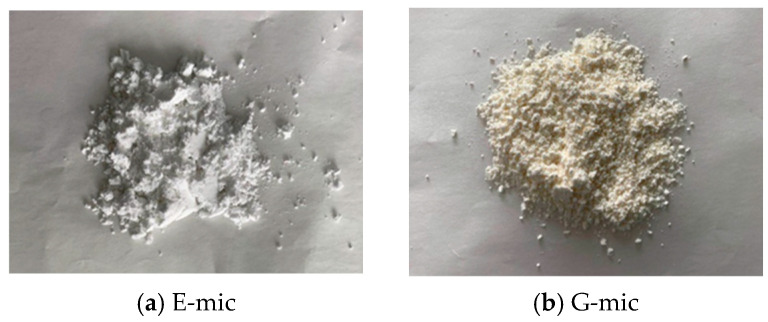
Microcapsule samples.

The microcapsules prepared in the laboratory are in the form of a dry powder, where the E-mic is pure white and the G-mic is milky white, as shown in Figure 7. Both types of microcapsule powders exhibit a uniform particle dispersion without obvious agglomeration, which is closely related to the in situ polymerization process adopted. During preparation, the core materials are first dispersed into micron-sized droplets by emulsifiers. The droplets are then modified to carry positive charges via the addition of the 0.5 wt% cationic surfactant. Subsequently, the shell precursor undergoes in situ polymerization on the surface of positively charged core droplets due to electrostatic adsorption, forming microcapsules with a core–shell structure. Finally, dry sieving yields powder products with an excellent dispersibility.

#### 3.1.1. Micromorphology and Microstructure Tests

**Figure 8:**
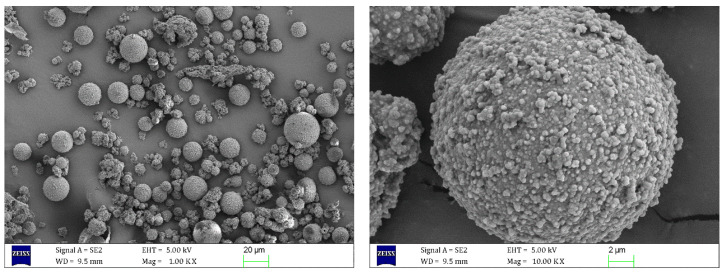
SEM images of E-mic.

**Figure 9:**
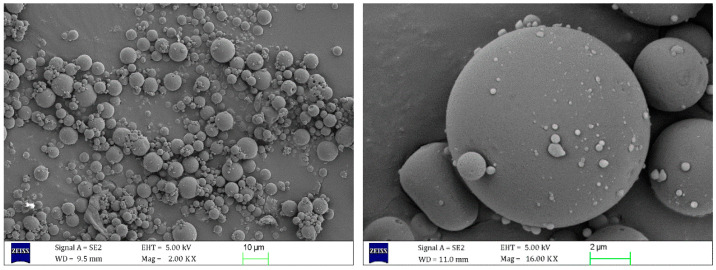
SEM images of G-mic.

The microstructure and morphology of the microcapsules were observed via SEM. Microscopic images are presented in Figure 8 and Figure 9.

As can be seen from Figure 8, the shape of the E-mic was close to spherical, with MUF particles adhering to its surface and a compact encapsulation. The relatively rough surface of the E-mic could improve its contact state with the matrix asphalt, making the modified asphalt system more stable. When asphalt sustains minor damage, epoxy resin E could flow out smoothly to repair the damage.

Figure 9 shows the micromorphology and microstructure of the G-mic. As depicted in Figure 9, the surface of the G-mic was slightly smoother than that of the E-mic. Similarly to the E-mic, the G-mic exhibited a good surface condition, with tightly packed and evenly distributed particles. This was conducive to the dispersion of microcapsules in asphalt, thereby enhancing the performance stability of the microcapsule-modified asphalt.

#### 3.1.2. Particle Size Analysis

**Figure 10:**
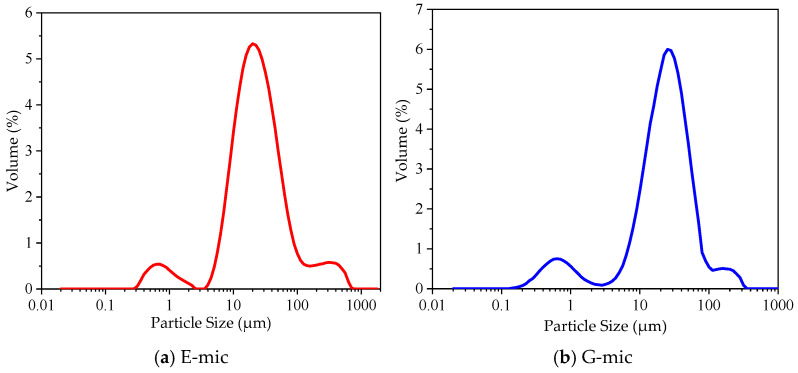
The particle size distribution of the microcapsules prepared.

The prepared microcapsules were powdery solids at the macroscopic level. The particle size analysis results of microcapsules are shown in Figure 10.

As shown in Figure 10a, the particle size distribution curve of the E-mic sample shows an approximately normal distribution, and the size is concentrated in a region ranging from 5 to 110 μm. Similarly to the E-mic, the particle size distribution curve of the G-mic sample also shows an approximately normal distribution, and the size is concentrated in a region ranging from 5 to 100 μm. The samples within each size range are analyzed, and the analysis results are obtained in Table 4.

As listed in Table 3, D(x) denotes the particles with a size smaller than D(x), which accounts for x% of the total number of particles, where x = 10, 50, and 90. Meanwhile, D50 also denotes the mean particle size. The consistency indicates the uniformity of the particle size distribution. The mean particle size of the E-mic sample was 23.582 μm, and the particle size of more than 90% was below 87.440 μm; the mean particle size of the G-mic sample was 22.440 μm, and the particle size of more than 90% was below 63.246 μm. From the particle size distribution test results, it can be seen that the particle size distributions of the two types of microcapsules are uniform, exhibiting good homogeneity.

#### 3.1.3. Microcapsule Thermal Stability Analysis

**Figure 11:**
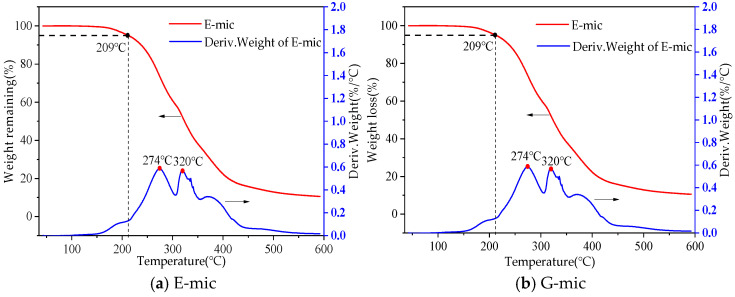
TGA curve for E-mic and G-mic.

The thermal stability of microcapsules was tested by a thermogravimetric analyzer. The TGA of the E-mic and G-mic is shown in Figure 11.

The TG curve represents the weight loss in the test, and the DTG curve is the derivative of the TG curve, that is, the change rate of the weight loss with the temperature. As shown in Figure 11a, the temperature at which the residual weight of the E-mic reaches 95% is 209 °C. This indicates that MUF begins to thermally decompose at 209 °C. When the temperature reaches 274 °C, the decomposition rate of MUF accelerates, indicating that at this temperature, the outer shell of MUF is essentially ineffective. As the temperature continues to rise to approximately 320 °C, a second peak appears on the DTG curve. Epoxy resin E also enters a rapid decomposition stage. As shown in Figure 11b, for the G-mic, the initial decomposition temperature is 223 °C, the first peak of the DTG is 275 °C, and the second peak is 382 °C.

Therefore, the E-mic and G-mic encapsulated by MUF have a high-heat resistance and can survive at high temperatures during asphalt construction without decomposition.

#### 3.1.4. Analysis of Interaction Mechanism Between Microcapsules and Asphalt

**Figure 12:**
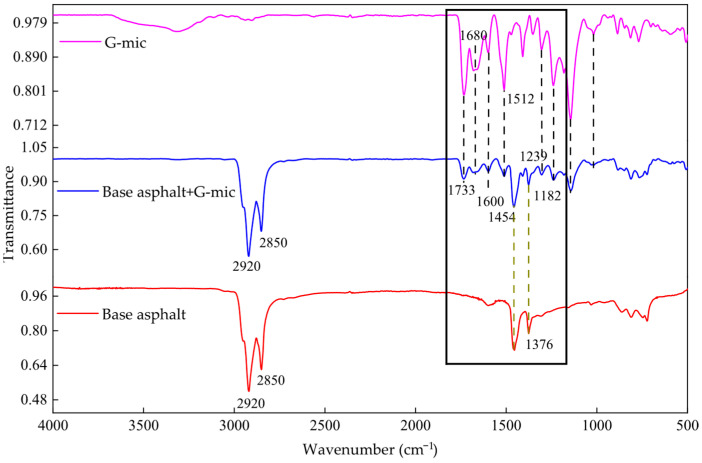
The infrared spectrum of the base asphalt and the base asphalt + G-mic.

The infrared spectrum of a substance is a reflection of its molecular structure, and the absorption peak in the spectrum corresponds to the vibration form of each group in the molecule. Figure 12 shows the corresponding infrared spectra of the G-mic after it is added to the base asphalt. The characteristic absorption peaks of C-H and C=O are near 2920 cm^−1^ and 2850 cm^−1^. The stretching vibration characteristic peak of C=O (in ester, aldehyde, ketone, acid) is near 1733 cm^−1^; the stretching vibration peak of C=C is near 1680 cm^−1^; the characteristic peak of the vibration absorption of the benzene ring is near the wavenumber of 1600 cm^−1^ is. The characteristic peaks of the asymmetric variable angle vibration and the symmetrical variable angle vibration of CH_3_ are near 1454 cm^−1^ and 1376 cm^−1^, respectively. The characteristic peak of the C-N stretching vibration is near 1239 cm^−1^ in the G-mic. The anti-symmetric stretching characteristic peak of the ether bond (C-O-C) in the G-mic is near 1182 cm^−1^. Based on the above analysis, this indicates that the G-mic is physically mixed with the asphalt, but there is no chemical reaction between them.

**Figure 13:**
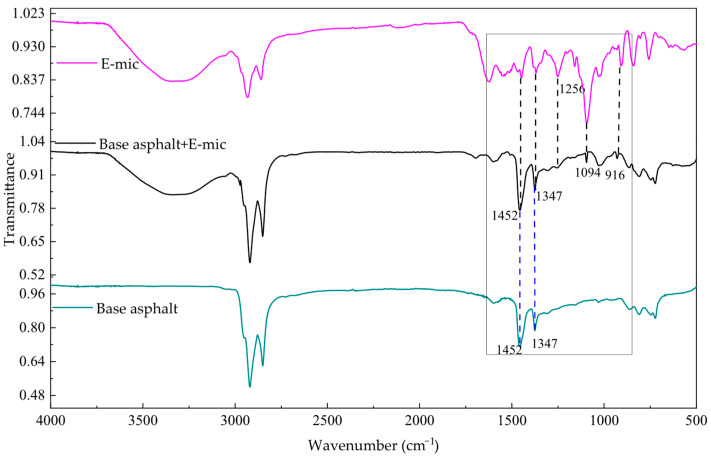
The infrared spectrum of the base asphalt and the base asphalt + E-mic.

Figure 13 shows the corresponding infrared spectra of the E-mic added to the base asphalt. Compared with the G-mic absorption peak, the N-H bending vibration absorption peak of the E-mic at 1500 is weakened, and the epoxy functional group is added at 916. This is related to the different functional groups of E-mic and G-mic molecules. The G-mic is a mixed modified amine system curing agent microcapsule, so the N-H bending vibration absorption peak is strong. The E-mic is an epoxy resin microcapsule, so it has an epoxy group absorption peak. In addition, there are strong vibrations near 1452 cm^−1^, which is the asymmetric variable angle vibration absorption peak of CH_3_ in the base asphalt and E-mic, while its symmetrical bending vibration absorption peak is near 1347 cm^−1^. The characteristic peak of the C-N stretching vibration is near 1256 cm^−1^ in the E-mic. The characteristic peak near 1094 cm^−1^ is the anti-symmetric stretching vibration absorption peak of C-C, and the characteristic absorption peak of the epoxy group is near 916 cm^−1^. Therefore, no new characteristic peaks appeared after the E-mic was added to the base asphalt. It can be concluded that the E-mic was physically mixed with the asphalt, but there is no chemical reaction.

### 3.2. High-Temperature Rheological Analysis

**Figure 14:**
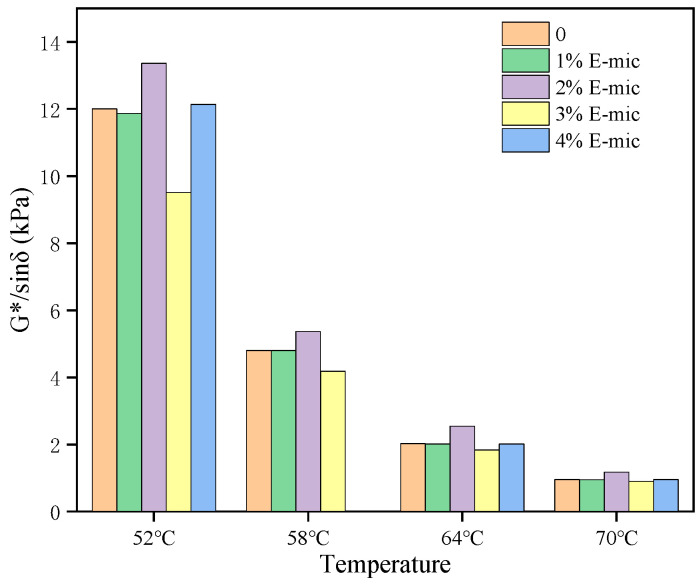
G*/sin δ of microcapsule/asphalt.

The ability of asphalt to resist permanent deformation at high temperatures has a very direct impact on the high-temperature rutting resistance of asphalt mixtures. SHRP proposed that the high-temperature performance of asphalt is characterized by the PG grading system, and the main evaluation indicator is the rutting factor (G*/sin δ). The rutting factor changes with the temperature after the TS tests of the microcapsule/asphalt are shown in Figure 14. G*/sin δ represents the ability of asphalt to resist permanent deformation at high temperatures. That is to say, the larger the value of G*/sin δ, the smaller the deformation of the asphalt under high temperatures and loads.

Figure 14 shows that, compared with that of the base asphalt, the G*/sin δ of the 2%E-mic is increased by 11.27%, 11.72%, 25.83, and 23.28%, respectively, at four temperatures. Compared with the base asphalt, the G*/sin δ of the 3%E-mic is reduced by 20.77%, 12.84%, 9.18%, and 5.62%, respectively, at four temperatures. The G*/sin δ of the 1% E-mic at all temperatures is smaller than that of the base asphalt, and the 4%E-mic is slightly larger than that of the base asphalt. The shell material of the E-mic is a thermosetting resin material, which is not sensitive to temperature and has a good elasticity. Therefore, the greater the dosage is, the greater the overall elasticity of the microcapsule asphalt material, while it is also affected by its liquid resin core material. Therefore, the greater the dosage of microcapsules, the greater the overall elasticity of the microcapsule/asphalt material will be. However, the overall mechanical properties of the microcapsules/asphalt are also influenced by the liquid epoxy resin core material. When the microcapsule/asphalt material is at a low temperature (52 °C), the elasticity of the 1% E-mic is enhanced, and the viscosity is reduced compared with the base asphalt, so the resistance to high-temperature deformation is reduced. As the dosage increases, the G*/sin δ reaches its peak at an E-mic dosage of 2% and its minimum at an E-mic dosage of 3%. When the microcapsule/asphalt material is at a higher temperature (70 °C), the G*/sin δ of the 1% E-mic, 3% E-mic, and 4% E-mic is less than 1.0 kPa. It can be speculated that the 2% E-mic and 4% E-mic have a better resistance to high-temperature deformation at the four temperatures.

### 3.3. Low-Temperature Crack Resistance Analysis

**Figure 15:**
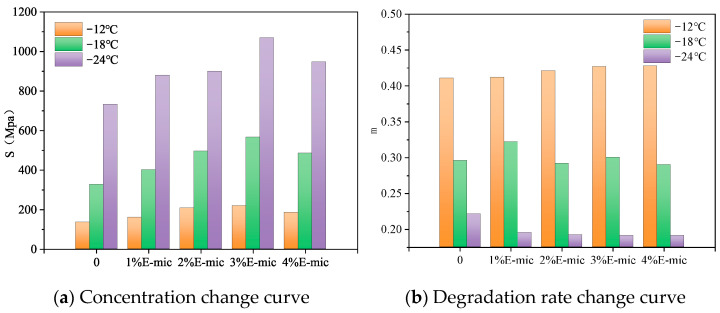
The results of the low-temperature creep of microcapsule/asphalt tests.

The low-temperature creep of microcapsule/asphalt tests was conducted by BBR and obtained the creep stiffness modulus (S) and its change rate (m) of different asphalt samples. A lower S and higher m indicate that the asphalt has good creep recovery characteristics at low temperatures. The test results are shown in Figure 15.

**Figure 16:**
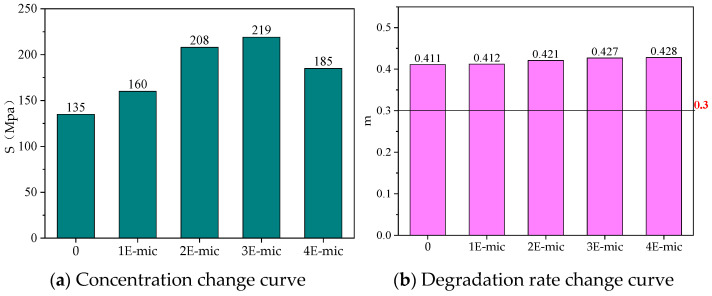
The results of the low-temperature creep of microcapsule/asphalt tests at −12 °C.

Figure 15 shows that at different temperatures, the stiffness modulus of the microcapsule/asphalt first increases and then decreases with increasing dosages. It indicates that the addition of microcapsules could increase the stiffness modulus of the asphalt. The more microcapsules that are added, the more shell materials exist in the microcapsule/asphalt. Therefore, the brittleness of the microcapsule/asphalt material increases, S increases, and m decreases. When the dosage of the microcapsule reaches 3%, S reaches the maximum. At −12 °C, the low-temperature crack resistance and stress relaxation of all test specimens are better. The creep stiffness modulus and its change rate of all samples at −12 °C are plotted in Figure 16.

As shown in Figure 16a, after adding the microcapsules, the stiffness modulus of the base asphalt was significantly increased. The stiffness modulus of microcapsule samples containing 1%, 2%, 3%, and 4% increased by 18.5%, 54.1%, 62.2%, and 37.0%, respectively. Although S increased by a large amount after the addition of the microcapsules, they still met the crack resistance requirements recommended by SHRP.

As shown in Figure 16b, the change rate of the stiffness modulus of the microcapsule samples containing 1%, 2%, 3%, and 4% increased by 0.2%, 2.4%, 3.9%, and 4.1%, respectively. After microcapsules were added, there was little change in m, and the m value of the modified asphalt was basically close to that of the base asphalt. Thus, it could be concluded that the addition of microcapsules did not significantly improve the relaxation performance of asphalt under low-temperature stress.

### 3.4. Fatigue Resistance Analysis

Each parameter of the LAS test corresponding to each sample was calculated based on the VECD theory.

(1) The Stress–Strain Analysis

**Figure 17:**
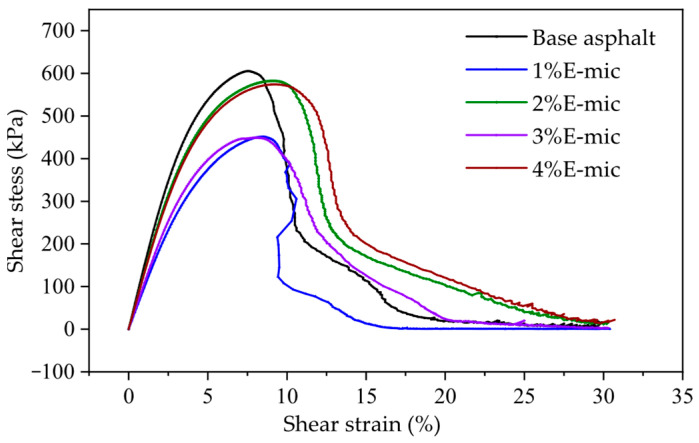
The stress–strain curve during the LAS test.

The stress–strain curve during the LAS test is plotted in Figure 17. As can be seen from Figure 17, the base asphalt and microcapsule/asphalt demonstrate a yield phenomenon during fatigue loading. At low strain levels, the shear stress of all samples increases rapidly with the increase in the shear strain level. Afterwards, peaks appear in the range of the 8%~12% strain. The range of this region represents the sensitivity of the sample to strain under certain shear actions. The wider the peak area, the lower the sensitivity and the higher fatigue performance; otherwise, the fatigue performance of the asphalt sample decreases. When the shear strain increases and the shear stress decreases, the asphalt samples are considered to have begun to suffer fatigue failure. Within the peak area, stress is not sensitive to the change in strain. After the peak area, the stress of all the asphalt samples decreases rapidly with the increase in strain. Except that the width of the peak area of the 1% E-mic was the same as that of the base asphalt, the peak width of other samples basically increased with the increase in the microcapsule dosage. The order of the strain level corresponding to the maximum shear stress of each sample is the 4% E-mic, 2% E-mic, 1% E-mic, 3% E-mic, and base asphalt. The larger the yield strain of each sample indicates the stronger the anti-fatigue performance of the asphalt mixture. The results show that the yield stress of all microcapsule/asphalt samples is greater than that of the base asphalt. Based on the above analysis, therefore, the addition of microcapsules can effectively improve the fatigue performance of the base asphalt.

(2) Fatigue Damage Analysis

**Figure 18:**
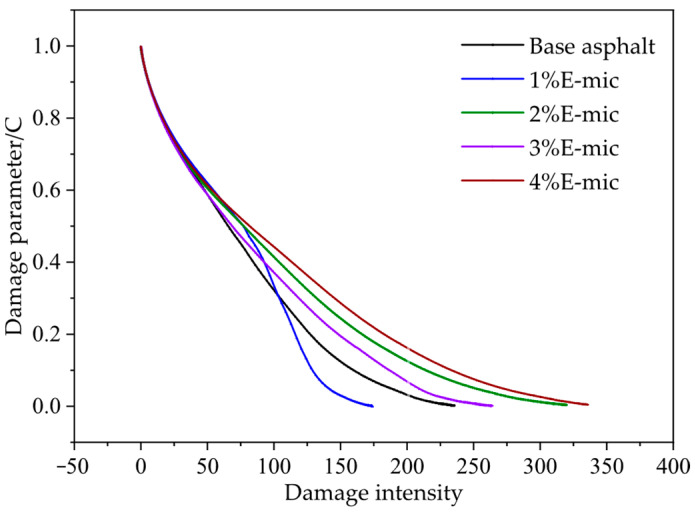
Fatigue damage curve.

The fatigue damage characteristic curve of asphalt samples is plotted in Figure 18. As shown in Figure 18, when the damage intensity is less than 100, the cumulative fatigue damage (C) decreases rapidly. When the damage intensity (D) is in the range of 100–200, the change rate of C slows down and presents a basically linear decrease. Afterwards, when D is in the range of 200–300, the change rate of C flattens out. Finally, the change rate of C does not increase when D exceeds 300. The change rate of the damage parameter C of asphalt samples can reflect their fatigue life. The greater the rate, the shorter the fatigue life; otherwise, the fatigue life increases. When D is in the range of 100–200, except for the 1% E-mic, the change rate of C for other samples is lower than that of the base asphalt. That is, the corresponding fatigue life will be better.

(3) Fatigue Life Analysis

**Figure 19:**
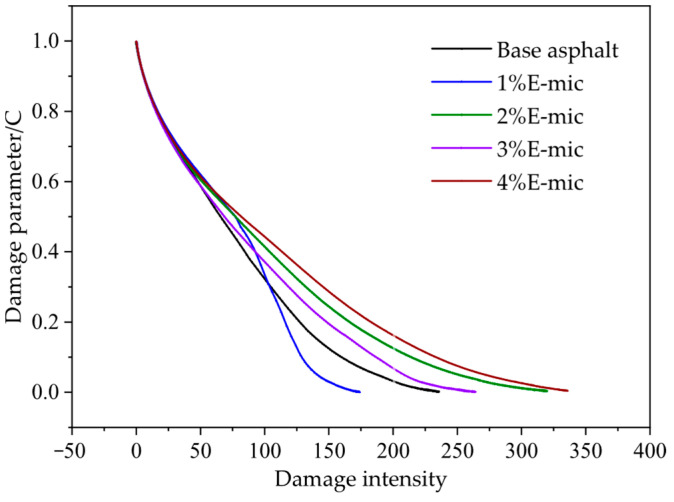
Fatigue life at different strain levels.

The influence of different strain levels on the fatigue performance of asphalt was analyzed and calculated. The fatigue life of the three strain levels, 2.5%, 5% and 10%, was calculated. The calculation results are shown in Figure 19.

As the strain level increases, the fatigue life of asphalt samples significantly decreases. When the strain level is 2.5%, the fatigue life of the base asphalt is 1.2 × 10^5^ times; when the strain level is 5%, the fatigue life is 968 times; and when the strain level increases to 10%, the fatigue life is only 76 times. When the strain level is 2.5%, the fatigue life of the 4% E-mic increases by 49.8% compared to that of the base asphalt. When the strain level is 5%, the fatigue life of the 2% E-mic and 4% E-mic significantly increased. Compared with the base asphalt, the fatigue life increases by 20.3% and 49.8%, respectively. When the strain level continues to increase to 10%, the fatigue life sequence is 4% E-mic > 2% E-mic > 3% E-mic > 1% E-mic > base asphalt. Therefore, microcapsules can effectively improve the fatigue performance of the base asphalt under high strain levels, and the 4% E-mic also has a better fatigue performance under low strain levels.

### 3.5. Self-Healing Property Analysis

**Figure 20:**
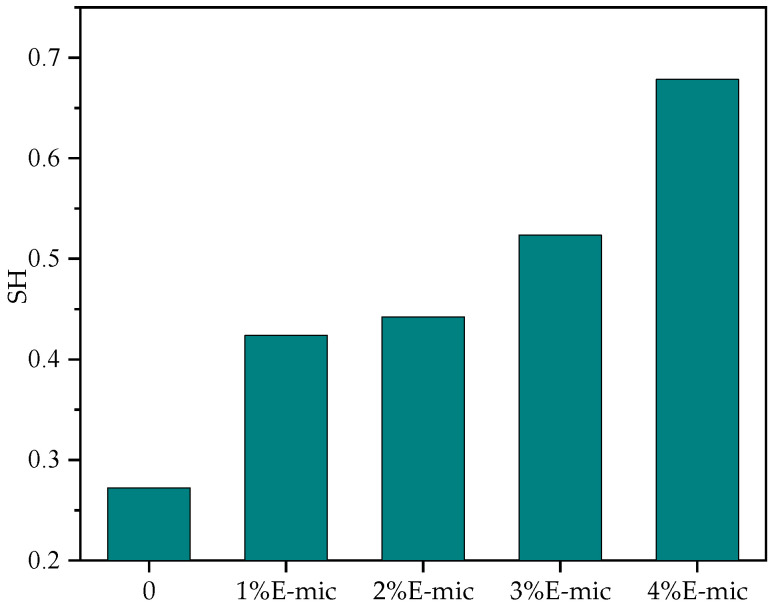
Self-healing rate of microcapsule asphalt with different dosages.

The influence of adding microcapsules on the self-healing property of asphalt with fatigue damage was evaluated through the fatigue repair fatigue test. Figure 20 shows the test results with different dosages.

As shown in Figure 20, the SH of the base asphalt is significantly improved by microcapsules, and with the increase in the microcapsule dosage, the self-healing rate of the asphalt gradually increases. When the dosage was 4%, SH reached a maximum value of 67.8%, which is 149.2% higher than that of the base asphalt. The greater the dosage of the microcapsule, the better the self-healing effect of the asphalt. It can be observed that when micro-cracks appear in asphalt, the more microcapsules that are used, the more core material they release. Meanwhile, more cured epoxy resin that can repair the asphalt matrix will be produced. Therefore, with the increase in the microcapsule dosage, the self-healing performance of asphalt is significantly improved.

### 3.6. The Recommended Dosage of the Microcapsule

Based on the aforementioned analysis, the optimal dosage of microcapsules can be recommended. The TS test showed that the deformation resistance of microencapsulated modified asphalt was better at dosages of 2% and 4%. Low-temperature bending creep tests indicated that the stiffness modulus increased after the addition of the microcapsules but still met the crack resistance requirements recommended by SHRP. LAS tests showed that the microcapsules could effectively improve the resistance to fatigue of the base asphalt, and the resistance to fatigue was better when the dosage was 2% and 4%. According to the self-healing performance test, as the microcapsule content gradually increases, the self-healing performance of the asphalt is significantly improved, with a better dosage of 4%. The comparison results are shown in Table 5. In conclusion, considering the performance and production cost of the microcapsule-modified asphalt, it is recommended that the optimal dosage of microcapsules be 2%.

This study prepared two-component microcapsules (E-mic and G-mic) using modified epoxy resin E and curing agent G as core materials, with their basic properties (e.g., particle size distribution, thermal stability) characterized. On this basis, modified asphalt samples with varying microcapsule dosages were prepared, and their pavement performance and self-healing capability were systematically evaluated to determine the optimal dosage range. Existing research on microcapsule-modified asphalt has predominantly focused on asphalt’s self-healing performance, with relatively little attention paid to the pavement performance of the modified asphalt. To address this gap, this study, following the successful preparation of weather-resistant two-component epoxy resin microcapsules, focuses on investigating the pavement performance of microcapsule-modified asphalt and reveals the influence of the microcapsule dosage on key asphalt properties, such as high-temperature resistance to permanent deformation and low-temperature rheological properties. The results indicate that the incorporation of two-component epoxy resin microcapsules enhances the asphalt’s high-temperature performance, self-healing ability, and fatigue life, while exerting adverse effects on the low-temperature performance. These findings are applicable to two-component epoxy resin microcapsule-modified asphalt. Notably, this paper rarely elaborates on the mechanism underlying the performance enhancement of microcapsule-modified asphalt. Additionally, it has not yet addressed the performance evolution law of the modified asphalt after repair, nor has it explored the aging resistance of microcapsule-modified asphalt [33]. These are all areas that require further improvement in subsequent research.

## 4. Conclusions

Two-component microcapsules (E-mic and G-mic) were prepared in this study. Subsequently, the microcapsules prepared were added to the base asphalt to prepare the microcapsules/asphalt material. Afterwards, temperature scanning (TS) tests, low-temperature bending creep tests, and linear amplitude scanning (LAS) tests were conducted to evaluate the road performance of the microcapsule/asphalt. Finally, the self-healing property tests were performed to evaluate the self-healing property of the microcapsule/asphalt. The conclusions of this study are as follows.

1. For spherical micron-sized particles with a uniform shape and particle size, the average particle size of the E-mic was 23.582 μm, and that of the G-mic was 22.440 μm, showing a good normal distribution. In addition, they could maintain good stability at high temperatures.
2. The TS tests indicated that the rutting resistance of the base asphalt gradually decreases with the increase in the temperature, while the high-temperature deformation resistance showed a better performance when the microcapsule dosage was 2% and 4%.
3. The low-temperature bending creep stiffness test demonstrated that the addition of microcapsules increased the stiffness modulus of the base asphalt, but the creep rate was not obvious. At −12 °C, the low-temperature crack resistance and stress relaxation of all specimens were better. The microcapsule can slightly improve the stress relaxation at low temperatures.
4. The stress–strain curve of LAS shows that yield stress is greater than that of base asphalt except when the microcapsule dosage is 3%. The fatigue damage curve of the LAS shows that at the range of 100–200, the microcapsule/asphalt fatigue damage rate is lower than that of the base asphalt, except for the microcapsule dosage of 1%; that is, the corresponding fatigue life will be better. The microcapsules can effectively improve the anti-fatigue performance of the base asphalt at high strain levels, and microcapsules/asphalt with a microcapsule dosage of 4% also have a higher anti-fatigue performance at low strain levels.
5. Considering the high-temperature deformation resistance, the low-temperature crack resistance, the fatigue resistance, and the self-healing property of asphalt, the optimal dosage of the microcapsules is recommended to be 4%.

## Figures and Tables

**Table 1:** The indicators of SK 70.

Indicators	Unit	Measured Value	Specified Value
Penetration (25 °C, 100 g, 5 s)	0.1 mm	66.7	60–80
Ductility (5 cm/min)	cm	>100	≥100
Softening point	°C	46.7	≥42/43
Viscosity (135 °C)	mPa s	475	-

**Table 2:** Main technical indicators of epoxy resin E.

Viscosity/mPa·s/25 °C	Epoxy Equivalent/ g·eq^−1^	Color/Max, G	Performance Overview
2300	215	1	Obtained by hydrogenation modification of bisphenol A type, colorless and transparent, weather-resistant, and yellowing-resistant.

**Table 3:** Raw materials for MUF preparation.

Material	Molecular Formula	Specification
Melamine (M)	C_3_N_3_(NH_2_)_3_	AR
Urea (U)	CH_4_N_2_O	AR
Formaldehyde (F)	CH_2_O	AR

**Table 4:** The results of the particle size analysis.

Samples	D(10) (μm)	D(50) (μm)	D(90) (μm)	Span	Consistency	Shading Degree
E-mic	7.096	23.582	87.440	1.113	0.343	10.93%
G-mic	4.477	22.440	63.246	1.157	0.355	12.90%

**Table 5:** Performance comparison of modified asphalt with different E-mic contents.

Content	Rutting Factor (64 °C)/kPa	Creep Stiffness Modulus (−12 °C)	Change Rate (−12 °C)	Self-Healing Rate
0	2.02	135	0.411	0.27
1	2.02	160	0.412	0.42
2	2.55	208	0.421	0.44
3	1.84	219	0.427	0.52
4	2.02	185	0.428	0.67

## Data Availability

The original contributions presented in this study are included in the article. Further inquiries can be directed to the corresponding author.

## Abbreviations

The following abbreviations are used in this manuscript:

E	Modified epoxy resin
G	Curing agent
MUF	Melamine modified urea–formaldehyde resin
E-mic	Modified epoxy resin microcapsule
G-mic	Curing agent microcapsule
